# Fibrin-Induced Epithelial-to-Mesenchymal Transition of Peritoneal Mesothelial Cells as a Mechanism of Peritoneal Fibrosis: Effects of Pentoxifylline

**DOI:** 10.1371/journal.pone.0044765

**Published:** 2012-09-13

**Authors:** Cheng-Chung Fang, Jenq-Wen Huang, Ren-Shi Shyu, Chung-Jen Yen, Cheng-Hsiang Shiao, Chih-Kang Chiang, Rey-Heng Hu, Tun-Jun Tsai

**Affiliations:** 1 Department of Emergency Medicine, National Taiwan University Hospital and National Taiwan University College of Medicine, Taipei, Taiwan; 2 Department of Internal Medicine, National Taiwan University Hospital and National Taiwan University College of Medicine, Taipei, Taiwan; 3 Department of Internal Medicine, Min-Sheng General Hospital, Taoyuan, Taiwan; 4 Department of Pathology, National Taiwan University Hospital and National Taiwan University College of Medicine, Taipei, Taiwan; 5 Department of Surgery, National Taiwan University Hospital and National Taiwan University College of Medicine, Taipei, Taiwan; University of Sao Paulo Medical School, Brazil

## Abstract

Excessive fibrin deposition in the peritoneum is thought to be involved in the development of encapsulating peritoneal sclerosis (EPS), an important cause of morbidity and mortality in peritoneal dialysis patients. We investigated fibrin-induced epithelial-to-mesenchymal transition (EMT) of peritoneal mesothelial cells (PMCs) as a possible mechanism of fibrin involvement in EPS. *In vitro*, fibrin overlay of PMCs altered their morphology; increased α-smooth muscle actin, fibronectin, fibroblast specific protein-1, and α_v_β_3_ integrin expression; and decreased cytokeratin 18 and E-cadherin expression. Fibrin overlay also increased focal adhesion kinase and Src kinase phosphorylation. Fibrin-induced changes were inhibited by treating the cells with α_v_β_3_ integrin antibody or pentoxifylline (PTX). In a rat model, intraperitoneal injection of *Staphylococcus aureus* and fibrinogen induced severe EPS features, which were attenuated by PTX treatment. PTX-treated rats also showed preserved peritoneal ultrafiltration function and lower concentrations of cytokines than the untreated rats. *S. aureus*- and fibrinogen-injected rats had higher percentage of cytokeratin-positive cells in the omentum fibrotic tissue than controls; this was also reduced by PTX treatment. Our results suggest that fibrin induces EMT of PMCs by engaging α_v_β_3_ integrin and activating associated kinases. Our EPS animal model showed that fibrin-induced EMT was involved in the pathogenesis of peritoneal fibrosis and was inhibited by PTX.

## Introduction

Long-term treatment with peritoneal dialysis (PD) can lead to progressive increase in extracellular matrix deposition and neovascularization [1]–[3]. These changes, called simple peritoneal fibrosis, affect the peritoneum as a dialysis organ; result in functional changes in the peritoneal membrane [4]; and subsequently necessitate discontinuation of PD due to ultrafiltration (UF) failure [5]. The most severe form of peritoneal fibrosis is encapsulating peritoneal sclerosis (EPS), a diffuse process involving the formation of new fibrous tissue that encapsulates the small bowel and constricts bowel motility [6]. EPS is an important cause of morbidity and mortality of PD patients, especially chronic PD patients [7], [8].

EPS pathogenesis is not clearly understood, but it can be hypothesized that persistent inflammation due to recurrent bacterial peritonitis or continuous exposure of the peritoneal membrane to bioincompatible dialysate is a prominent cause of EPS [9]. Excessive fibrin deposition on the peritoneal membrane is also associated with EPS [9]. When the peritoneum is injured, bleeding and leakage of plasma proteins from damaged surfaces form a fibrinous deposit in the abdominal cavity. Subsequently, the fibrin mesh is invaded by proliferating fibroblasts, which replace the fibrin with more durable components of the extracellular matrix [10]. Normally, the fibrinolytic system activation results in lysis of these intra-abdominal fibrin deposits; however, if this mechanism fails, fibrous adhesions are formed [11]. Imbalance between intraperitoneal fibrin formation and removal may be a key component involved in the pathogenesis of EPS [12]. Although excessive fibrin formation may be involved in EPS [13], to date, fibrin has been considered only as an adhesive agent that binds tissues.

Although epithelial-mesenchymal transition (EMT) is well studied in embryonic development, it also participates in fibroblast genesis during organ fibrosis in adult tissues [14]. Yanez-Mo et al. [15] have shown that peritoneal mesothelial cells (PMCs) undergo EMT during PD. Furthermore, EMT plays an important role in peritoneal fibrosis resulting from PD [16] and is related to high peritoneal transport in PD [17], [18]. In the present study, we investigated whether EMT is responsible for the effects of fibrin in peritoneal fibrosis. We studied the effects of fibrin on PMCs *in vitro* and established an animal model of EPS by intraperitoneally injecting the animals with *S. aureus* and fibrinogen and examined the impact of excessive fibrin formation on peritoneal fibrosis. Furthermore, we investigated *in vitro* and *in vivo* the effects of treatment with pentoxifylline (PTX), a xanthine derivative inhibiting proliferation and collagen synthesis of PMCs and fibroblasts [19], [20].

## Results

### Fibrin induces EMT of human PMCs

**Figure 1:**
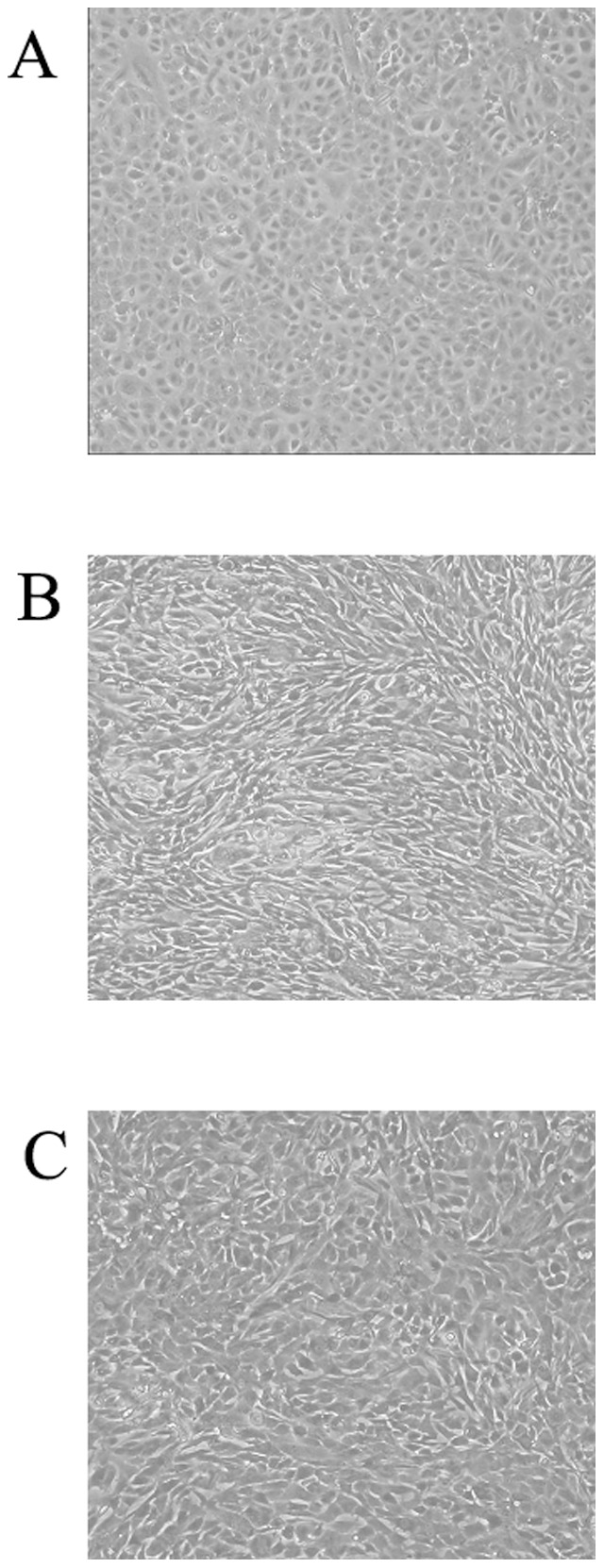
Morphological changes in peritoneal mesothelial cells (PMCs) after fibrin application. The morphology of the PMCs changed from a polygonal cobblestone-like appearance (A) to a spindle-shaped form (B) after fibrin overlay for 24 h. (C) Morphological changes were attenuated by treatment with 0.3 mg/ml of pentoxifylline.

**Figure 2:**
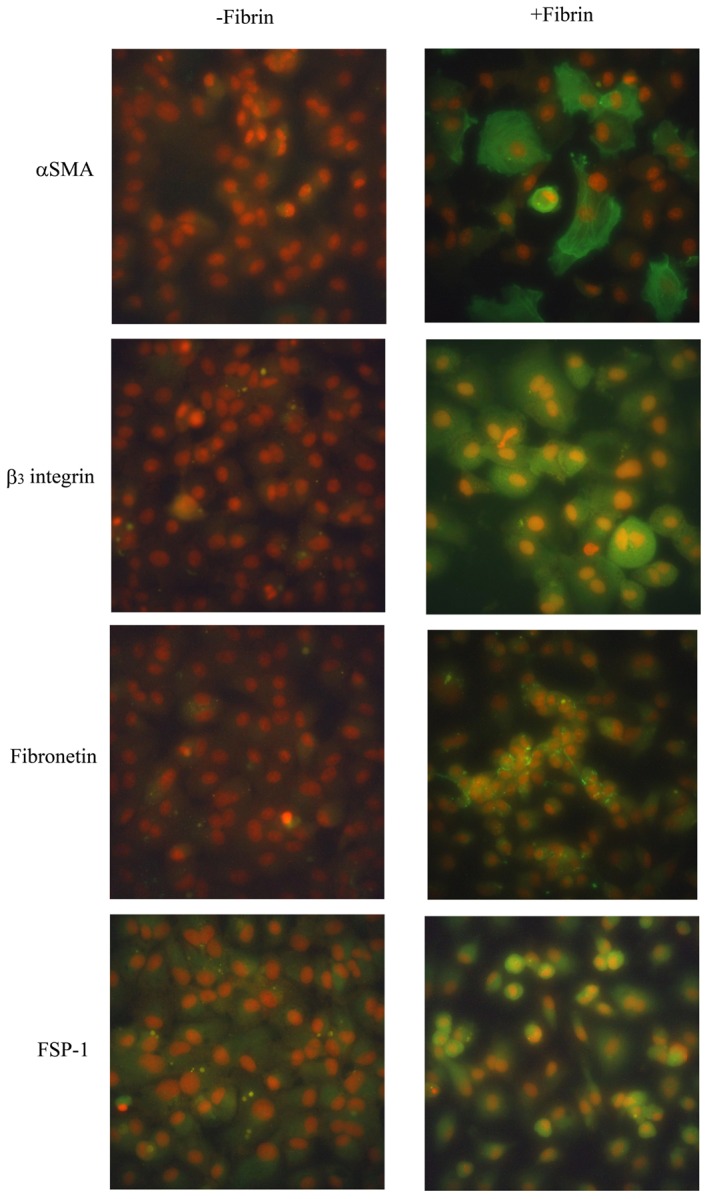
Changes in cell markers after application of fibrin to peritoneal mesothelial cells (PMCs). Expression of α-smooth muscle actin (α-SMA), fibronectin, fibroblast specific protein-1 (FSP-1), and β_3_ integrin were detected by immunofluorescence staining with FITC-labeled secondary antibodies (green). Nuclei were counterstained with PI (red). PMCs overlaid with fibrin for 4 h (+Fibrin) expressed higher levels of α-SMA, fibronectin, FSP-1, and β_3_ integrin than untreated PMCs (-Fibrin). Original magnification ×400.

**Figure 3:**
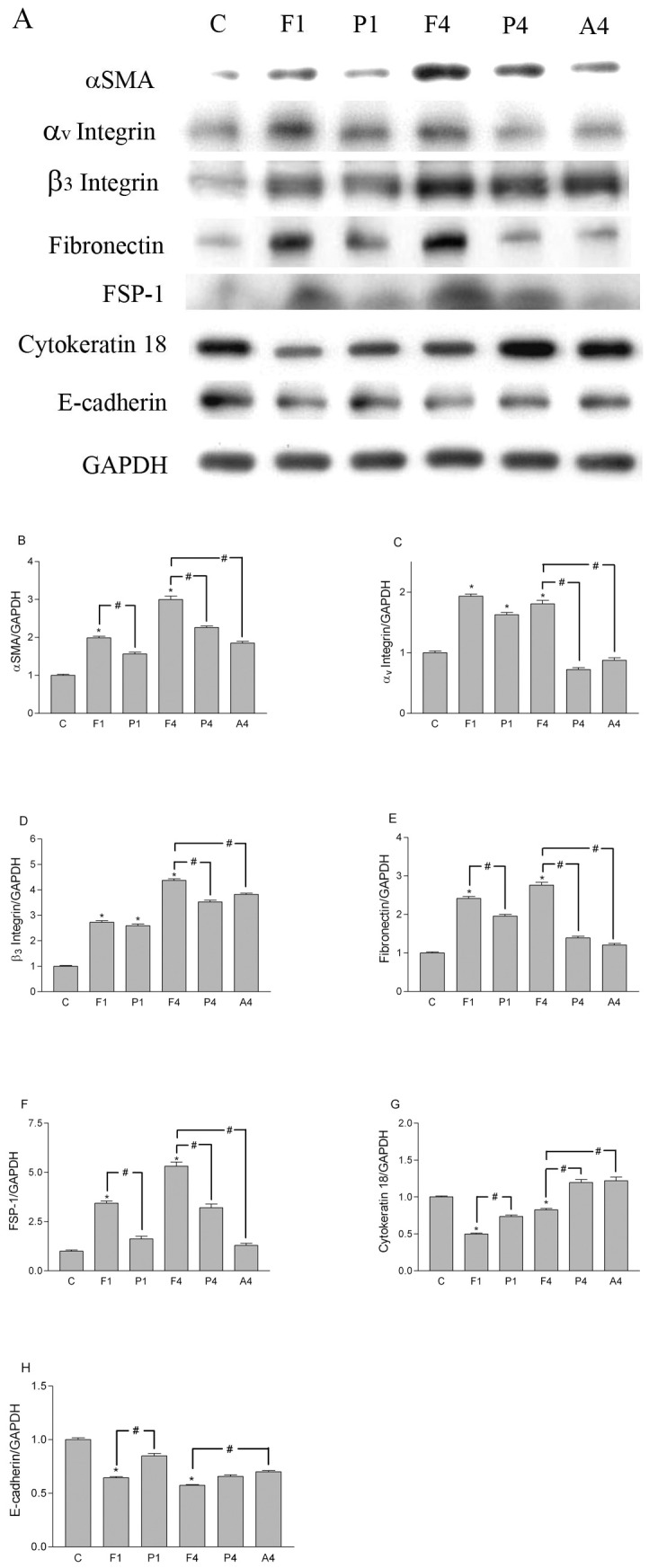
Western blots of cell markers in fibrin-covered peritoneal mesothelial cells (PMCs). Equal amounts of protein (20 µg per lane) from untreated or fibrin-covered PMCs were resolved, transferred, and blotted for α-SMA, fibronectin, FSP-1, α_v_β_3_ integrin, cytokeratin 18, E-cadherin, and GAPDH (A). The relative levels of α-SMA/GAPDH (B), α_v_β_3_ integrin/GAPDH (C and D), fibronectin/GAPDH (E), FSP-1/GAPDH (F), cytokeratin 18/GAPDH (G), and E-cadherin/GAPDH (H) were measured by densitometry. C, PMCs without fibrin; F1, fibrin covered for 1 h; P1, fibrin covered and treated with pentoxifylline (PTX) 0.3 mg/ml for 1 h; F4, fibrin covered for 4 h, P4, fibrin covered and treated with PTX 0.3 mg/ml for 4 h; A4, fibrin covered and treated with α_v_β_3_ integrin antibody for 4 h. *P<0.05 vs. C, # P<0.05 between groups, n = 3.

Cultures of human PMCs were overlaid with fibrin by incubating with a mixture of fibrinogen and thrombin for 24 h. During that time, the morphology of the PMCs changed from a polygonal cobblestone-like appearance (Fig. 1A) to spindle-shaped (Fig. 1B). These morphological changes were attenuated by the treatment of 0.3 mg/ml PTX (Fig. 1C). Cells cultured for 4 h were examined by immunofluorescence for expression of α-smooth muscle actin (α-SMA), fibronectin, fibroblast specific protein-1 (FSP-1) and β_3_ integrin. PMCs overlaid with fibrin expressed higher levels of all proteins, compared with untreated PMCs (Fig. 2). These observations were confirmed by western blotting of transformed cells 1 h and 4 h after overlay with fibrin (Fig. 3). In contrast, the expression of cytokeratin 18 and E-cadherin decreased after PMCs were covered by fibrin (Fig. 3). Of note, the fibrin-induced changes in expression of α-SMA, fibronectin, FSP-1, α_v_β_3_ integrin, cytokeratin 18, and E-cadherin were all attenuated after treatment of PMCs with either PTX or an α_v_β_3_ integrin antibody (Fig. 3).

**Figure 4:**
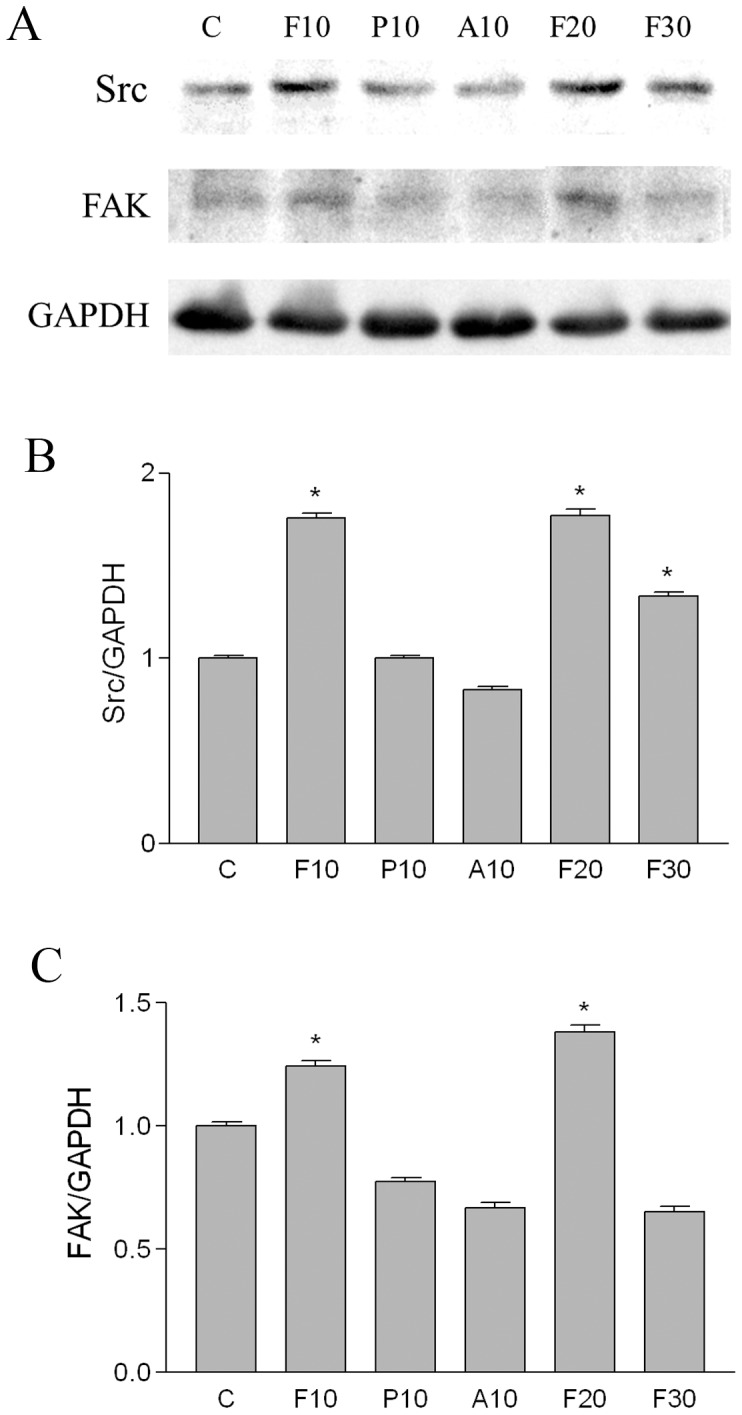
Western blots of integrin-activated kinases. Equal amounts of protein (20 µg per lane) from untreated or fibrin-covered PMCs were resolved, transferred, and probed for phosphorylated FAK (focal adhesion kinase) and Src (A). The relative levels of Src/GAPDH (B) and FAK/GAPDH (C) were measured by densitometry. C, PMCs without fibrin; F10, fibrin covered for 10 min; P10, fibrin covered and treated with pentoxifylline 0.3 mg/ml for 10 min; A10, fibrin covered and treated with α_v_β_3_ integrin antibody for 10 min; F20, fibrin covered for 20 min; F30, fibrin covered for 30 min. *p<0.05 vs. Control.

To determine the intracellular signaling pathways that might mediate the effects of fibrin on PMCs, we performed western blotting to examine phosphorylation of 2 kinases commonly activated through integrins; FAK (focal adhesion kinase) and Src. As shown in Fig. 4, phosphorylation of both FAK and Src was increased after fibrin application and was attenuated by treatment with PTX or α_v_β_3_ integrin antibody. These data suggest that fibrin-induced EMT of PMCs is mediated through α_v_β_3_ integrin-stimulated activation of Src and FAK, and is inhibited by PTX.

### Animal model of EPS

**Figure 5:**
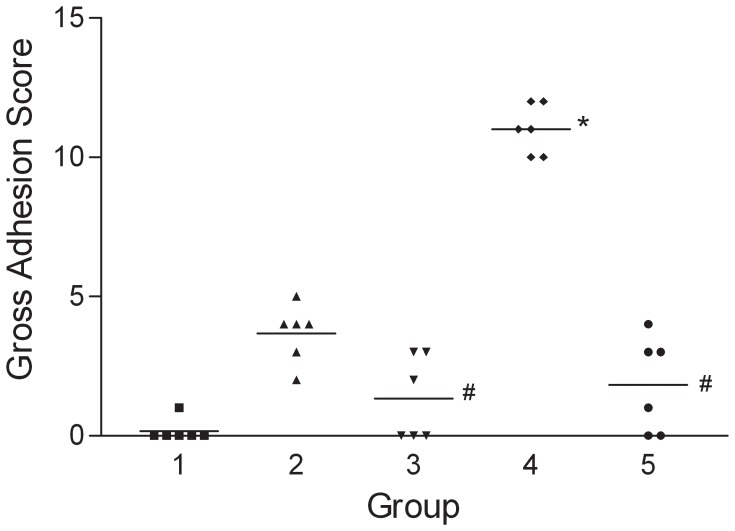
Gross adhesion scores. Group 4 had a significantly higher score than Group 1. Group 5 had a significantly lower score than Group 4. *p<0.05 vs. Group 1, #p<0.05 vs. Group 4.

**Figure 6:**
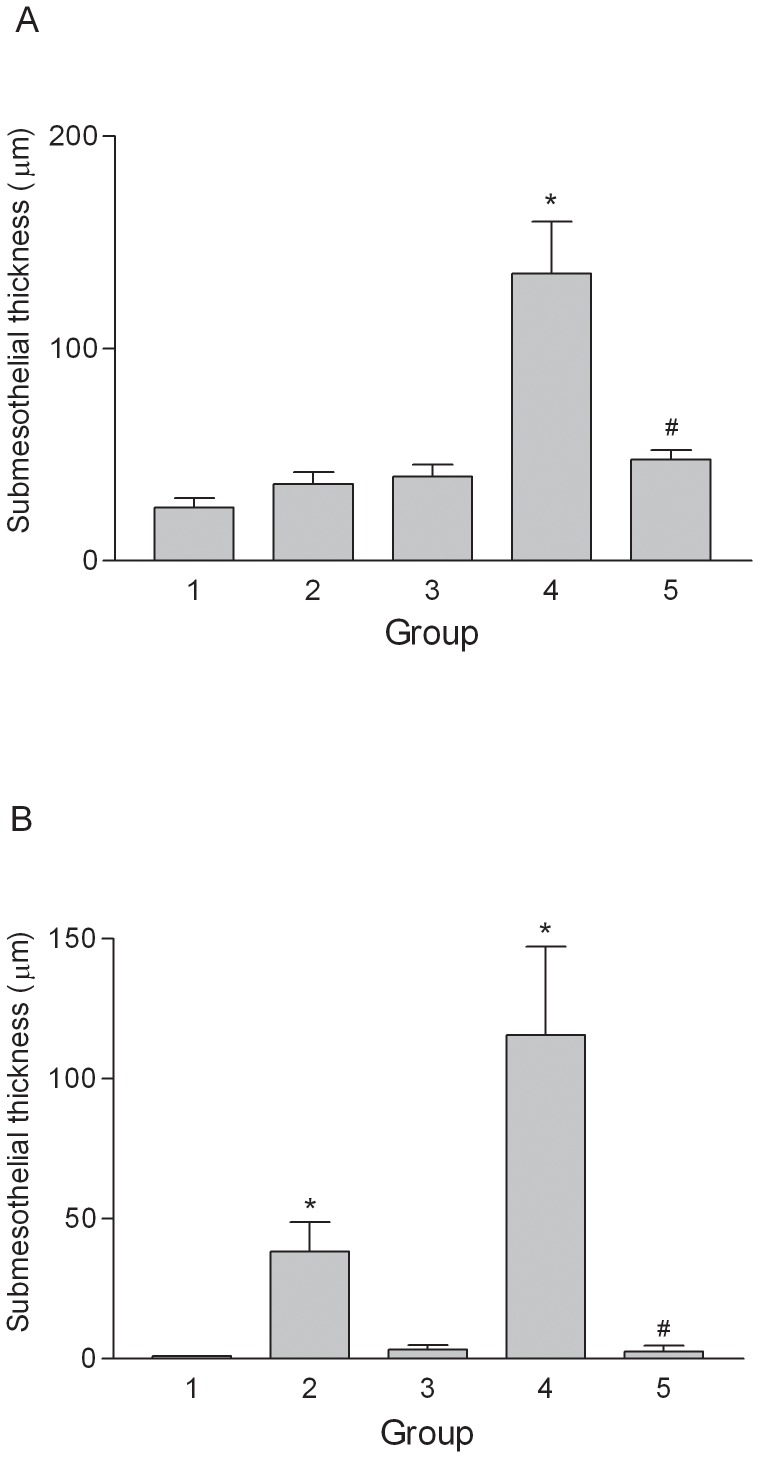
Histological analysis of the submesothelial compact zones. The compact zones were thicker in Group 4 than in Group 1 on both the parietal peritoneum (A) and liver surface (B). Rats treated with PTX (Group 5) had significantly less submesothelial matrix formation than Group 4 on both the parietal peritoneum and liver surface. *p<0.05 vs. Group 1 (Control), #p<0.05 vs. Group 4.

To determine if fibrin-induced EMT might participate in the pathogenesis of EPS and if PTX could have therapeutic effects on EPS, we established a rat model of EPS by intraperitoneal injection with *Staphylococcus aureus* and fibrinogen. Animals injected with both *S. aureus* and fibrinogen (Group 4, supporting figure S1) displayed more severe features of EPS than did the rats injected with either *S. aureus* (Group 2) or fibrinogen (Group 3) alone. As shown in Fig. 5, the intraperitoneal gross adhesion scores were significantly higher for Group 4 than for the untreated control animals in Group 1. Of note, daily administration of PTX to animals injected with both *S. aureus* and fibrinogen resulted in significantly lower gross adhesion scores (Group 5 vs. Group 4; Fig. 5). As shown in Fig. 6, animals with severe EPS had thicker submesothelial compact zones than control animals (Group 4 vs. Group 1) in both the parietal peritoneum and liver surface. Rats injected with *S. aureus* alone demonstrated increased submesothelial compact zone thickness on the surface of the liver compared with the control animals (Group 2 vs. Group 1). In contrast, rats treated concomitantly with PTX (Group 5) showed significantly less submesothelial matrix formation than Group 4 animals on both the parietal peritoneum and liver surfaces.

Peritoneal function was evaluated by a 2-h peritoneal equilibrium test (PET). As shown in Table 1, peritoneal permeability to urea and creatinine was similar among the treatment groups. However, permeability to albumin and total protein were greater in Groups 2, 3, and 4 than in Group 1. Both albumin and total protein permeability were significantly lower for rats in Group 5 compared with Group 4. Similarly, absorption of glucose from the dialysate was significantly greater in Groups 2 and 4 than in Group 1, but was significantly less in Group 5 than in Group 4. The mean peritoneal volume at the end of PET was significantly smaller for Groups 2 and 4 than Group 1 (Table 1). Moreover, the mean peritoneal volume at the end of PET for Groups 2 and 4 (18.8±0.7 ml and 17.6±0.8 ml) were smaller than the initial infusion volume (20 ml), indicating a loss in UF function. Treatment of animals with PTX significantly improved UF function, as illustrated by the increase in peritoneal volume at the end of PET in Group 5 (21.1.±0.7 ml) compared with Group 4 (17.6±0.8 ml).

**Table 1:** Peritoneal equilibrium tests.

	Group 1 (Control)	Group 2 (B only)	Group 3 (F only)	Group 4 (B+F)	Group 5 (B+F+PTX)
Albumin (D2/S)	0.010±0.002	0.034±0.004*	0.050±0.012*	0.112±0.014*	0.013±0.004†
Total protein (D2/S)	0.003±0.003	0.057±0.011*	0.084±0.025*	0.141±0.014*	0.027±0.007†
Urea (D2/S)	0.87±0.01	0.96±0.02	0.86±0.03	0.96±0.02	0.93±0.03
Creatinine (D2/S)	0.77±0.05	0.83±0.06	0.75±0.07	0.65±0.05	0.71±0.07
Sugar (D2/D0)	0.290±0.013	0.169±0.012*	0.282±0.016	0.114±0.012*	0.262±0.019†
Dialysate volume (ml)	23.4±0.7	18.8±0.7*	22.8±0.7	17.6±0.8*	21.1±0.7†

Data are expressed as mean ± SEM. D2, dialysate after 2 h dwell; D0, dialysate at the beginning of peritoneal equilibrium test; S, serum; PBS, phosphate buffered saline; B, bacteria; F, fibrinogen; PTX, pentoxifylline.

* P<0.05 vs. Group 1,

† P<0.05 vs. Group 4.

The concentrations of cytokines in the dialysate at the end of PET were measured by ELISA and are provided in Table 2. The concentrations of vascular endothelial growth factor (VEGF) and transforming growth factor β (TGFβ) were significantly higher in Group 4 than in Group 1, and were significantly lower in Group 5 compared with Group 4. The concentrations of interleukin-1β (IL-1β), tumor necrosis factor α (TNFα) and interleukin-6 (IL-6) were significantly higher in Groups 2 and 4 than in Group 1, and were also significantly lower in Group 5 compared with Group 4. The concentration of plasminogen activator inhibitor type 1 (PAI-1) was significantly higher in Groups 2, 3, and 4 than in Group 1, and was significantly lower in Group 5 compared with Group 4. The concentration of tissue plasminogen activator (tPA) was significantly higher in Groups 2, 3, 4 and 5 compared with Group 1, but there were no significant differences among Groups 2, 3, 4, and 5.

**Table 2:** Cytokine concentrations in peritoneal dialysates.

	Group 1 (Control)	Group 2 (B only)	Group 3 (F only)	Group 4 (B+F)	Group 5 (B+F+PTX)
VEGF (pg/ml)	2.7±0.8	11.1±2.0	12.0±2.4	93.2±16.2*	10.0±1.8†
TGFβ (pg/ml)	111±21	206±28	118±16	1277±275*	216±38†
IL-1β (pg/ml)	27±11	149±21*	34±8	381±38*	87±10†
TNFα (pg/ml)	371±76	1880±160*	302±69	2530±526*	470±135†
IL-6 (pg/ml)	235±50	1780±242*	380±93	4850±1230*	418±4†
PAI-1 (ng/ml)	13±2	108±13*	53±9*	175±26*	21±4†
tPA (pg/ml)	5±1	79±15*	48±11*	118±23*	56±13*

Data are expressed as mean ± SEM. PBS, phosphate buffered saline; B, bacteria; F, fibrinogen; PTX, pentoxifylline.

* P<0.05 vs. Group 1,

† P<0.05 vs. Group 4.

**Figure 7:**
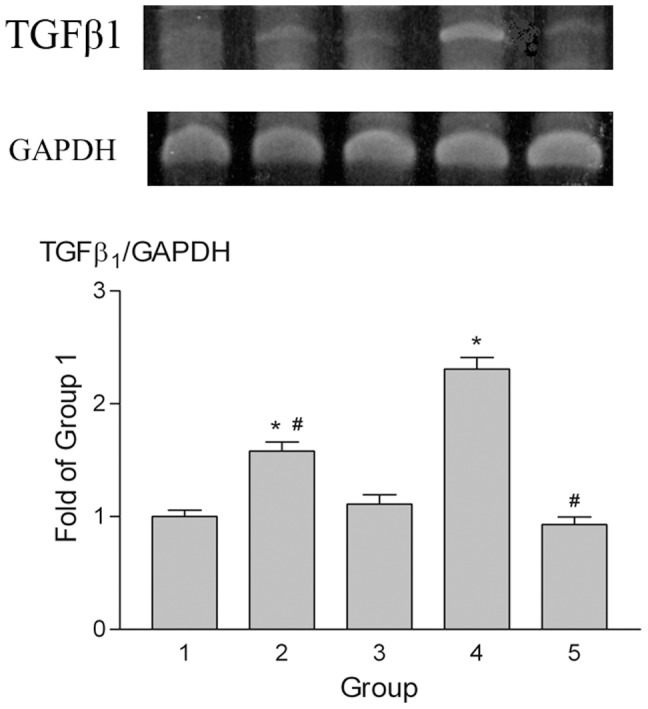
TGFβ1 gene expression in the parietal peritoneum. TGFβ1 mRNA was measured by RT-PCR. mRNA levels increased in Groups 2 and 4 compared to Group 1 but expression in Groups 2 and 5 was significantly lower than in Group 4. GAPDH expression served as a loading control. *p<0.05 vs. Group 1, #p<0.05 vs. Group 4.

TGFβ1 gene expression in the parietal peritoneum was examined by RT-PCR (Fig. 7). The results show that TGFβ1 mRNA levels were increased in Groups 2 and 4 animals compared with those in Group 1 and were significantly lower in Groups 2 and 5 than in Group 4.

**Figure 8:**
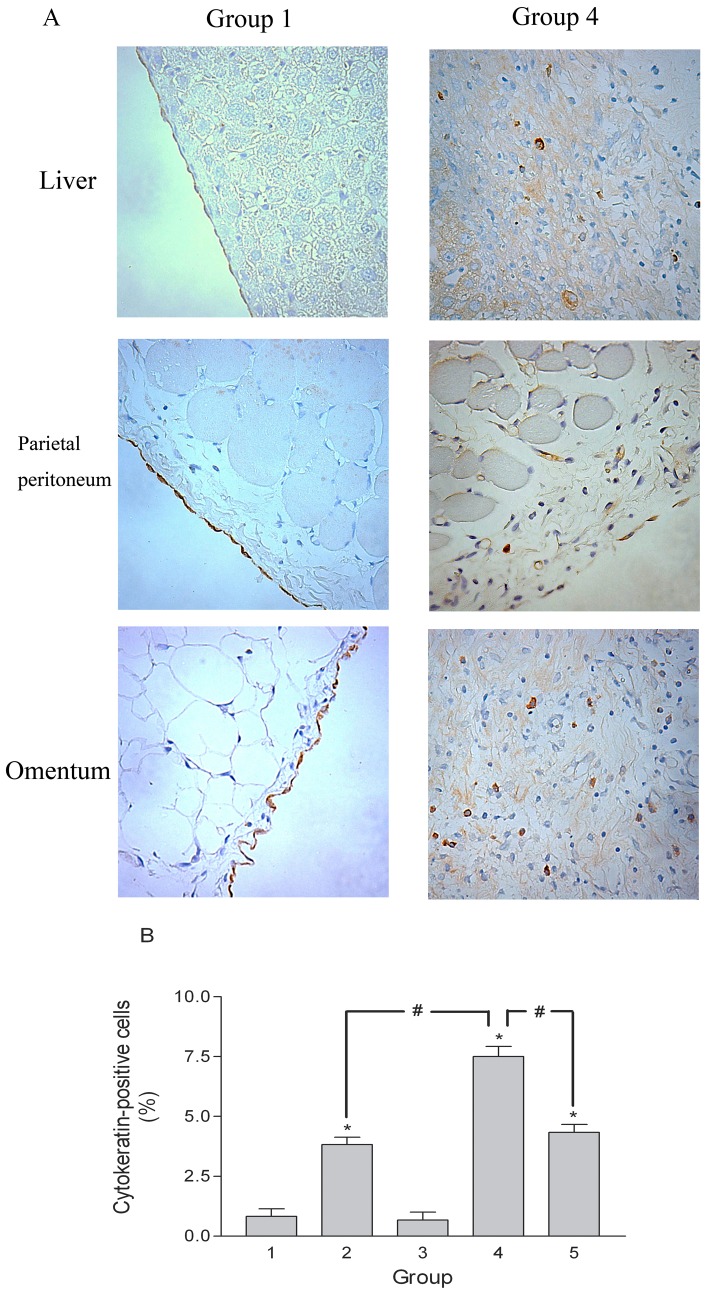
Immunohistochemical staining of cytokeratin. (A) Cytokeratin-positive cells were present not only on the surface mesothelium, but also in the submesothelial fibrosis on the liver surface, parietal peritoneum, and omentum of Group 4 rats. Cytokeratin-positive cells were present only on the surface mesothelium of Group 1 rats. Original magnification ×400. (B) Percentage of cytokeratin-positive cells in the fibrotic tissue of the omentum. *p<0.05 vs. Group 1, #p<0.05.

**Figure 9:**
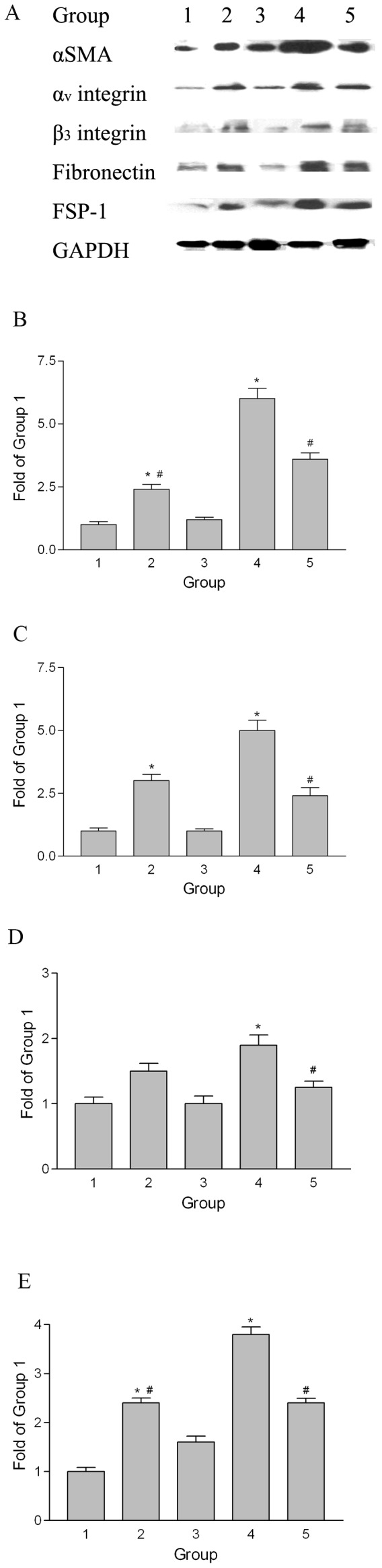
Western blots of cell markers in the peritoneum of animals with EPS. Equal amounts of protein (30 µg per lane) from the peritoneum of rats in Group 1 to 5 were resolved, transferred, and probed for α-SMA, α_v_β_3_ integrin, fibronectin, FSP-1, and GAPDH (A). The relative levels of α-SMA/GAPDH (B), α_v_β_3_ integrin/GAPDH (C and D), fibronectin/GAPDH (E), and FSP-1/GAPDH (F) were measured by densitometry.*p<0.05 vs. Group 1, #p<0.05 vs. Group 4.

To demonstrate that the PMCs had undergone EMT in this animal model, we performed immunohistochemical staining for cytokeratin. In the samples from Group 4 rats, cytokeratin-positive cells were found not only on the surface mesothelium but also in submesothelial fibrotic tissue on the liver surface, parietal peritoneum, and omentum (Fig. 8A). However, cytokeratin-positive cells were only detected on the surface mesothelium in Group 1 rats (Fig. 8A). The percentages of cytokeratin-positive cells in the fibrotic tissue of omentum were much higher in rats of Groups 2, 4, and 5 than in control animals (Group 1), and significantly higher in Group 4 than in Groups 2 or 5 (Fig. 8B). These findings provide evidence that EMT is occurring in this animal model of peritoneal fibrosis, and that excessive fibrin promotes EMT *in vivo*. Moreover, these changes are inhibited by treatment of animals with PTX. The cell markers changes in peritoneum of animal are shown in Figure 9. The markers (α-SMA, fibronectin, FSP-1 and α_v_β_3_ integrin) increased in the peritoneum of the Group 4 rats and could be ameliorated by PTX treatment.

## Discussion

Although excessive fibrin formation has been proposed as a key component of EPS development [13], [21], the mechanism of action of fibrin in EPS has not been studied previously. In this study, we demonstrated that fibrin-covered PMCs transformed into myofibroblast-like cells, as indicated by changes in cell morphology and expression of cell markers (increased α-SMA, fibronectin, FSP-1, and α_v_β_3_ integrin, and decreased E-cadherin and cytokeratin). These observations support the hypothesis that fibrin induces EMT of PMCs. We also showed that fibrin activated FAK and Src kinase activity in PMCs and that the fibrin-induced changes in expression of cell markers and kinase activity were attenuated by addition of an anti-α_v_β_3_ integrin antibody. These data support that the fibrin-induced EMT is mediated through the activation of the α_v_β_3_ integrin receptor, which would transduce into cell differentiation [22].

It is known that EMT plays an important role in the peritoneal fibrosis of PD patients [15], [16]. One potential therapeutic strategy for treatment of peritoneal fibrosis is to prevent the EMT of mesothelial cells [23]. We demonstrated the signaling pathway of PTX on PMCs is due to the phospodiesterase inhibitory property through activation of the cAMP/PKA pathway [20]. We also established that PTX inhibits the TGF-β-induced collagen gene expression in PMCs through modulating the ERK1/2 and p38^HOG^ pathways [24]. PTX has also been shown to inhibit renal fibrosis in a rat model of crescentic glomerulonephritis by affecting renal tubular EMT through blockade of TGF-β1 expression and Smad2/3 activation [25]. In this study, we validated that PTX decreased fibrin-induced EMT of PMCs through inhibiting fibrin-activated FAK and Src kinase. Therefore, PTX may have therapeutic effects on peritoneal fibrosis, and the PTX effects on our animal model of EPS were also verified in this research.

To determine if fibrin-induced EMT might participate in the pathogenesis of EPS and if PTX could have therapeutic effects on EPS, we established an animal model of EPS. Although many models have been proposed to induce EPS in animals [26], [21], none of these models accurately reflect the clinical situation and incorporate clinically relevant causative agents of EPS [26]. The pathogenesis of EPS has been hypothesized in a so-called two-hit-model [27]. The first hit damages the peritoneal mesothelium, leads to the production and secretion of cytokines (VEGF, TGF, etc.), and causes an enhancement of fibosis and angiogenesis. Additionally, the fibrinolytic activity is reduced because of the loss of mesothelial cells and mast cells. The second hit, which can be for example an inflammatory stimulus (like a bacterial peritonitis) takes place, EPS can develop. Myofibroblasts are further activated, more inflammatory, profibrogenic and angiogenic mediators are produced and additional fibrin is deposited [27]. Therefore, we chose to induce EPS in rats by bacterial peritonitis and intraperitoneal injection of fibrinogen, which are more clinically relevant stimuli. In keeping with this, injected animals exhibited similar pathological peritoneal changes to those observed in EPS in humans, both macroscopically and microscopically [13], [27]. Functionally, our model results in lower net UF and higher transport rates, which are the key features of PET results of patients with EPS [28]–[31]. Importantly, we found cytokeratin-positive cells in the submesothelial fibrotic tissues of affected rats, demonstrating that EMT was involved in this model of EPS, which is similar to the clinical presentation of EPS patients [32].

In this study, the features of EPS and cytokeratin-positive cells in the submesothelial fibrotic tissues were more prominent in rats with bacterial peritonitis plus intraperitoneal fibrinogen injection (Group 4) than in animals with bacterial peritonitis alone (Group 2). This might suggest that excessive fibrin deposition could induce EMT and influence EPS. However, intraperitoneal injection of fibrinogen alone (Group 3) induced only minimal pathology. This may be explained by the elevated tPA production of intact PMCs after intraperitoneal injection of fibrinogen. Bioincompatible dialysate stimulates PMCs to express PAI-1 [33]. Bacterial peritonitis induces many inflammatory cytokines that reduce the fibrinolytic ability of mesothelial cells by increasing PAI-1 production [34]. Both *in vitro* and *in vivo* [35], [36] studies have shown that the increase in PAI-1 synthesis, not the decrease in the production of tPA, alters mesothelial fibrinolytic activity. In addition, our results revealed that intraperitoneal PAI-1 increased in rats injected with both bacteria and fibrinogen. The increase in tPA activity in rats with peritonitis with or without fibrinogen injection may be explained by excessive fibrin formation. However, PAI-1 plays a key role in the development of fibrinolytic activity. Therefore, our data are consistent with the hypothesis that increased PAI-1 activity results in decreased fibrinolytic activity, which leads to excessive fibrin deposition and further triggers the development of EPS.

Progressive increases in peritoneal membrane permeability and UF failure are commonly observed in PD patients [5]. Neoangiogenesis induced by VEGF has been proposed as the cause of UF failure [37] and is known to play a critical role in an animal model of EPS [38]. In this study, we found that VEGF content was increased in the peritoneal effluent of animals injected with bacteria and fibrinogen. The PET results showed that animals with high VEGF levels had increased vascular permeability and UF failure. PMCs that have undergone EMT have been shown to be the main source of VEGF in PD patients and, therefore, may be responsible for the high peritoneal transport rate [17]. We demonstrated that fibrin-induced EMT is involved in this animal model of EPS and may be implicated in the excessive VEGF production and UF failure.

The therapeutic effect of PTX on EPS was also displayed in our animal model both macroscopically and microscopically. Moreover, PTX improved peritoneal function and suppressed the release of IL-1β, TNFα, IL-6, VEGF, and TGFβ. PTX has multiple effects on the immune system, including inhibition of IL-1β, TNFα, and IL-6 production through activation of the cAMP/PKA pathway and/or inhibition of NFκB [39]. PTX also enhances fibrinolytic activity by stimulating the release of tPA and plasmin [40]. Therefore, the therapeutic effects of PTX may be achieved through attenuation of EMT by PMCs, inhibition of cytokine production, and/or the promotion of fibrinolysis.

In conclusion, we have shown that fibrin induces EMT of PMCs through α_v_β_3_-stimulated FAK and Src signaling. We established an animal model of EPS demonstrating the involvement of fibrin-induced EMT in the pathogenesis of peritoneal fibrosis. We also demonstrated that PTX inhibited the fibrin-induced EMT and shows clinically relevant therapeutic benefit in this animal model of EPS.

## Methods

### 1. Ethics Statement

Peritoneal mesothelial cells were cultured from human omentum. Specimens of human omentum were obtained from patients undergoing gastrectomy due to early gastric carcinoma. Patients submitted written informed consent prior to the procedure, and the protocol was approved by the Research Ethics Committee of National Taiwan University Hospital. All rat experiments were performed under the American Association for Accreditation for Laboratory Animal Care regulations, and approved by the Institutional Animal Care and Use Committee of National Taiwan University College of Medicine.

### 2. Materials

PTX was purchased from Hoechst AG (Frankfurt am Main, Germany). All other chemicals were from Sigma (St. Louis, MO, USA) unless otherwise specified. The immunoassay kits for VEGF and TGFβ were purchased from R&D Systems, Inc. (Minneapolis, MN, USA). The immunoassay kits for IL-1β, TNFα, and IL-6 were purchased from Biosource (Camarillo, CA, USA). The immunoassay kit for PAI-1 was from American Diagnostica Inc. (Stamford, CT, USA). The immunoassay kit for tPA was from Innovative Research (Plymouth, MN, USA). Reagents for western blot analysis were from Bio-Rad Laboratories Inc. (Hercules, CA, USA). Mouse monoclonal antibody to α-SMA was purchased from Signet Laboratories (Dedham, MA, USA). Rabbit monoclonal antibody to FSP-1 was from Abcam plc. (Cambridge, UK). Mouse monoclonal antibody to E-cadherin was from Calbiochem (Gibbstown, NJ, USA). Mouse monoclonal antibodies to α_v_ and β_3_ integrin domains were from Becton-Dickinson (Franklin Lakes, NJ, USA). Rabbit monoclonal antibodies to phospho-FAK and phospho-Src were from Invitrogen Co. (Carlsbad, CA, USA). Rabbit monoclonal antibody to GAPDH, mouse monoclonal antibody to fibronectin, goat anti-mouse IgG and anti-rabbit IgG secondary antibodies were from Santa Cruz Biotechnology, Inc. (Santa Cruz, CA, USA).

### 3. Cell culture experiments

Enzymatic disaggregation of omentum was performed as previously described [20]. The human PMCs were initially bipolar or multipolar but became cobblestone-like in appearance upon confluence. PMCs were identified by immunofluorescence staining for the presence of vimentin and cytokeratin and the absence of desmin and factor VIII-related antigen. All experiments were performed using cells between passage 1 and 3 and were repeated at least 3 times using cells from different subjects.

The cytotoxic effects of fibrinogen (10 mg/ml in serum free RPMI), thrombin (0.2 U/ml) and fibrin clot (by mixing fibrinogen with thrombin) on PMCs were excluded by 3-[4,5-dimethylthiazol-2-yl]-2,5-diphenyltetrazolium test (supporting figure S2) [20]. PMCs were cultured in a 6-well plate to sub-confluence in RPMI 1640 medium supplemented with 10% fetal bovine serum (FBS). Fibrinogen, bovine serum albumin (BSA, 10 mg/ml in FBS free RPMI), thrombin and fibrin clot were added on top of the cells to evaluate the changes of cell proteins. Fibrin clot and fibrinogen increased α-SMA, β_3_ integrin and fibronectin expressions, whereas thrombin and BSA had no effects (supporting figure S3). Because the effects of fibrin clot were greater than fibrinogen alone and fibrin was proposed as one of the pathogenesis in peritoneal fibrosis, we used fibrin clot to study the EMT effect on PMCs in the following studies. Where relevant, PTX was added to the fibrin clot at a concentration of 0.3 mg/ml. The fibrin clots were removed at various times and the cells were lysed for protein extraction or prepared for immunofluorescence staining.

### 4. Immunofluorescence staining

PMCs were fixed with 4% paraformaldehyde, permeabilized with 0.2% Triton X-100, and blocked by incubation in 10% FBS in phosphate buffered saline (PBS). Cells were then incubated with primary antibodies to α-SMA, fibronectin, β_3_ integrin, or FSP-1, washed, and incubated with fluorescein isothiocyanate (FITC)-labeled goat anti-mouse or anti-rabbit IgG secondary antibodies, and propidium iodide was used to counterstain nuclei. The slides were observed under a fluorescence microscope.

### 5. Western blotting

PMCs were lysed in ice-cold lysis buffer (65 mM Tris base, pH 8.0, 154 mM NaCl, 1 mM EDTA, 1% IGEPAL, 1 mM PMSF, 1 µg/ml leupeptin, 1 µg/ml pepstatin, 1 µg/ml aprotinin, and 0.25% sodium deoxycholate). The samples were rotated for 15 min at 4°C and then centrifuged at 12,000 *g* for 5 min at 4°C. The supernatants were recovered and protein concentration was measured by the bicinchoninic acid assay (Bio-Rad) with a bovine serum albumin standard. Clarified lysates were incubated for 5 min at 95°C in loading buffer (12 mM Tris-HCl, pH 6.8, with 25% glycerol, 2% SDS, 14.4 mM 2-mercaptoethanol, and 0.1% bromophenol blue) and 20 µg of protein per lane were loaded on SDS-polyacrylamide gels of differing percentages, as appropriate for the molecular weights of the target proteins. After electrophoresis, the proteins were electrotransferred to polyvinylidene difluoride membranes, which were incubated in blocking buffer (1% BSA, 0.05% Tween in PBS) overnight at 4°C. Membranes were then incubated with primary antibodies to E-cadherin, cytokeratin 18, α-SMA, fibronectin, α_v_β_3_ integrin, FSP-1, phosphorylated FAK and Src, and GAPDH, followed by horseradish peroxidase-labeled goat anti-mouse or anti-rabbit IgG secondary antibodies. Blots were developed by incubation in a chemiluminescence substrate and exposure to X-ray film.

### 6. Animal model of encapsulating peritoneal sclerosis

**Figure 10:**
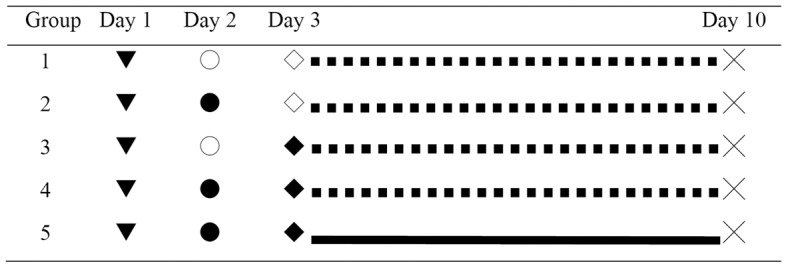
Protocol for the animal study. Thirty male Wistar rats were randomly divided into 5 groups of 6 rats per group. Rats received intraperitoneal injections of PBS (Group 1), *S. aureus* (6×10^8^ colony forming units (CFU); Group 2), 15 ml fibrinogen (10 mg/ml; Group 3), or both *S. aureus* and fibrinogen (Group 4). Group 5 animals received intraperitoneal injections of *S. aureus* and fibrinogen and also received daily intravenous injections of PTX (100 mg/kg) for 7 days. Animals were then subjected to the peritoneal equilibration test (PET) and sacrificed. The details of procedures are shown in the figure. ▾: Catheter placement in the internal jugular vein. ○: PBS intraperitoneally. •: 1 ml *S. aureu*s (6×10^8^ CFU/ml) intraperitoneally. ◊: 15 ml PBS intraperitoneally. ⧫: 15 ml fibrinogen (10 mg/ml) intraperitoneally. ▪ ▪ ▪ ▪ ▪: 1.5 ml intravenous PBS daily for 7 days. ▪▪▪▪▪▪: 100 mg/kg intravenous pentoxifylline (20 mg/ml; approximately 1.5 ml) daily for 7 days. ×: Performed PET and then sacrificed.

Thirty male Wistar rats (250–300 g) were given access to water and standard rat chow *ad libitum*. *Staphylococcus aureus* ATCC 25923 (American Type and Culture Collection, MD, USA) was used throughout the study. Before each experiment, a fresh stock was grown culture in Luria-Bertani broth (Invitrogen) for 8 h in an agitated water bath at 37°C. Pilot studies were performed to establish optimal bacterial and fibrinogen dosages, the time for initiation of PTX therapy, and the time of sacrifice. The animals were divided into 5 groups (n = 6) according to the experimental protocol shown in Fig. 10. Group 1 rats served as Control and receiving phosphate buffered saline (PBS) injection. Group 2 rats were injected with *S. aureus* (6×10^8^ colony forming units) intraperitoneally, while Group 3 rats were injected with 15 ml fibrinogen (10 mg/ml) intraperitoneally. Group 4 rats received both *S. aureus* and fibrinogen injections, and Group 5 rats received PTX treatment (0.1 mg/day/g of rat weight) after *S. aureus* and fibrinogen injections. The animals were injected with PTX or PBS intravenously for 7 days and then sacrificed after PET.

### 7. Evaluation of peritoneal function

Peritoneal function was evaluated by a 2-h PET as follows [41]. The animals were anesthetized by intravenous ketamine injection and peritoneal and internal carotid catheters were placed. The animals were then infused intraperitoneally with 20 ml of 2.5% Dianeal (Baxter International Inc., Deerfield, IL, USA) solution containing 1 mg/ml blue dextran. Dialysate samples (1 ml) were taken at time 0 (immediately after infusion) and again after 60 and 120 min for determination of concentrations of urea, creatinine, glucose, albumin, and total protein. Blood samples were taken from the internal carotid catheter at the beginning and end of the PET. The rats were anesthetized by ketamine for the duration of the PET. At the end of the PET, dialysate was drained from the peritoneal catheter and 20 ml of PBS was infused. Concentrations of IL-1β, TNFα, IL-6, VEGF, TGFβ, tPA, and PAI-1 in the dialysate samples were measured by immunoassay. The volume of residual dialysate was calculated from the dilution of blue dextran in the PBS wash, as previously described [41]. The total volume of dialysate at the end of PET was calculated as the sum of the drainage and residual volumes.

### 8. Morphometry of the peritoneum

The severity of peritoneal adhesions at the abdominal wall, intestine, omentum, testis, spleen, and liver was scored according to the chart provided in Table 3. Tissue samples from the liver and abdominal wall were collected to evaluate fibrosis at the submesothelial interstitium. Parietal peritoneum was obtained from the left upper part of the anterior abdominal wall and a sample of liver was taken from the right lobe. To avoid the effects of gross adhesion on microscopic examinations, these samples were extracted from the area least affected by adhesions. The samples were fixed in 10% buffered formaldehyde and embedded in paraffin.

**Table 3:** Gross adhesion scoring system.

Omentum	Score	Spleen	Score
Normal	0	Normal	0
Small (<5 mm) and single nodule	1	Irregular surface	1
Large (>5 mm) or multiple nodules	2	Adhesion with omentum	2
Ball formation	3	Completely encapsulated	3

Omentum-liver adhesion	Score	Intestine adhesion	Score
No adhesion	0	No adhesion	0
Mild adhesion (liver edge <10 mm)	1	Mild adhesion (single adhesion band)	1
Moderate adhesion	2	Moderate adhesion	2
Severe adhesion (whole liver edge)	3	Severe adhesion (>3 loops)	3

Omentum-parietal peritoneum adhesion	Score	Liver	Score
No adhesion	0	Normal	0
Mild adhesion (single adhesion band)	1	Uneven surface	1
Moderate adhesion	2	Small nodules or dull edge	2
Severe adhesion (>3 bands or >10 mm)	3	Fusion of lobes	3

Omentum-testis adhesion	Score	Parietal peritoneum	Score
No adhesion	0	Normal	0
One side	1	Uneven surface	1
Two sides	2	Small (<5 mm) and single nodule	2
		Large (>5 mm) or multiple nodules	3

Paraffin blocks were cut (5 µm-thick sections) and stained with hematoxylin and eosin or Masson's Trichrome stain by standard procedures. The submesothelial compact zone is stained blue by Masson's Trichrome stain (supporting figure S4). Each tissue section was measured at 5 points per microscopic field (magnification, ×100) and 5 fields were recorded. The average thickness of the submesothelial compact zone was then determined.

### 9. Immunohistochemistry

Immunohistochemistry for cytokeratin was performed using a mouse anti-cytokeratin antibody and development with diaminobenzidine (Invitrogen), according to the manufacturer's instructions. Cells within the submesothelial fibrotic regions of the omentum were counted in 20 high-power fields. The number of cytokeratin-positive cells was recorded for each sample and expressed as a percentage of the total number of cells.

### 10. Gene expression in the parietal peritoneum

The peritoneal surface of anterior abdominal wall sections was immersed in Trizol reagent for 15 min. Then the parietal peritoneum was gently scraped while Trizol was collected and processed according to the manufacturer's instructions [42]. Reverse transcription of 2.5 µg of total RNA was performed using oligo(dT) primers and reverse transcriptase (Promega, Madison, WI, USA), as directed. Amplification of TGFβ1 was performed for 28 cycles of 30 s at 94°C, 30 s at 58°C, and 1 min at 72°C with forward primer 5′-TCCACAGAGAAGAACTGCTG-3′ and reverse primer 5′-ACTTGCAGGAGCGCACAATC-3′. The housekeeping gene GAPDH was included as a positive control for each specimen and was amplified with forward primer 5′-GAGTCAACGGATTTGGTCGT-3′ and reverse primer 5′-TTCCCGTTCTCAGCCTTGAC-3′. PCR conditions for GAPDH were 25 cycles of 30 s at 94°C, 30 s at 55°C, and 1 min at 72°C, which was confirmed to be within the exponential phase. PCR products were electrophoresed on 1% TBE-PAGE gels and stained with ethidium bromide to visualize DNA bands.

### 11. Statistical analysis

All data are expressed as mean ± SEM. Comparison of gross adhesion scores was performed with non-parametric Kruskal-Wallis test. Analysis of other data from different animal groups was performed by one-way ANOVA with *post hoc* analysis. A P value less than 0.05 was considered significant.

## Supporting Information

Figure S1 **Gross morphology of the peritoneal cavity in a rat model of encapsulating peritoneal sclerosis.** Rats were sacrificed 8 days after injection of *S. aureus* and fibrinogen. A representative rat showing adhesions among the liver, omentum, and intestine. The liver surface was uneven with fusion between lobes.
(DOC)

Figure S2 **The Agents were excluded to have cytotoxic effect on PMCs.** Bovine serum albumin (BSA, 10 mg/ml), fibrinogen (10 mg/ml), thrombin (0.2 U/ml) and fibrin (by mixing fibrinogen with thrombin at the same concentration) were added into PMC culture for 48 hr. A 3-[4,5-dimethylthiazol-2-yl]-2,5-diphenyltetrazolium bromide (MTT) assay was performed as described (reference 20 in the article). All data are expressed as the means with SEM of 3 experiments conducted in pentaplicate. All agents did not show statistical difference vs. control.
(DOC)

Figure S3 **Western blots of cell markers in peritoneal mesothelial cells (PMCs) with various agents.** Equal amounts of protein (20 µg per lane) from untreated, bovine serum albumin, thrombin, fibrinogen, or fibrin treated PMCs for 4 hr were resolved, transferred, and blotted for α-SMA, fibronectin, β_3_ integrin, and GAPDH (A). The relative levels of α-SMA/GAPDH (B), β_3_ integrin/GAPDH (C), fibronectin/GAPDH (D) were measured by densitometry. C, PMCs without agent; BSA, bovine serum albumin 10 mg/ml; T, thrombin 0.2 U/ml; FG, fibrinogen 10 mg/ml; F, fibrinogen 10 mg/ml mixed with thrombin 0.2 U/ml. *P<0.05 vs. C, # P<0.05 between fibrinogen and fibrin, n = 3.
(DOC)

Figure S4 **Micrographs of tissues from rats injected intraperitoneally with** ***S. aureus*** **and fibrinogen.** The submesothelial compact zones of the parietal peritoneum (A) and liver (B) were stained blue with Masson's Trichrome stain. Each tissue section was measured at 5 points per microscopic field and 5 fields were recorded. The average thickness of the submesothelial compact zone was determined.
(DOC)
