# Supplementary material for: Fibrin-Induced Epithelial-to-Mesenchymal Transition of Peritoneal Mesothelial Cells as a Mechanism of Peritoneal Fibrosis: Effects of Pentoxifylline
Source: PLoS One. 2012 Sep 13;7(9):e44765. doi: 10.1371/journal.pone.0044765 (PMC3441450; doi:10.1371/journal.pone.0044765)

**Supporting figure S1. Gross morphology of the peritoneal cavity in a rat model of encapsulating peritoneal sclerosis.**

Rats were sacrificed 8 days after injection of *S. aureus* and fibrinogen. A representative rat showing adhesions among the liver, omentum, and intestine. The liver surface was uneven with fusion between lobes.


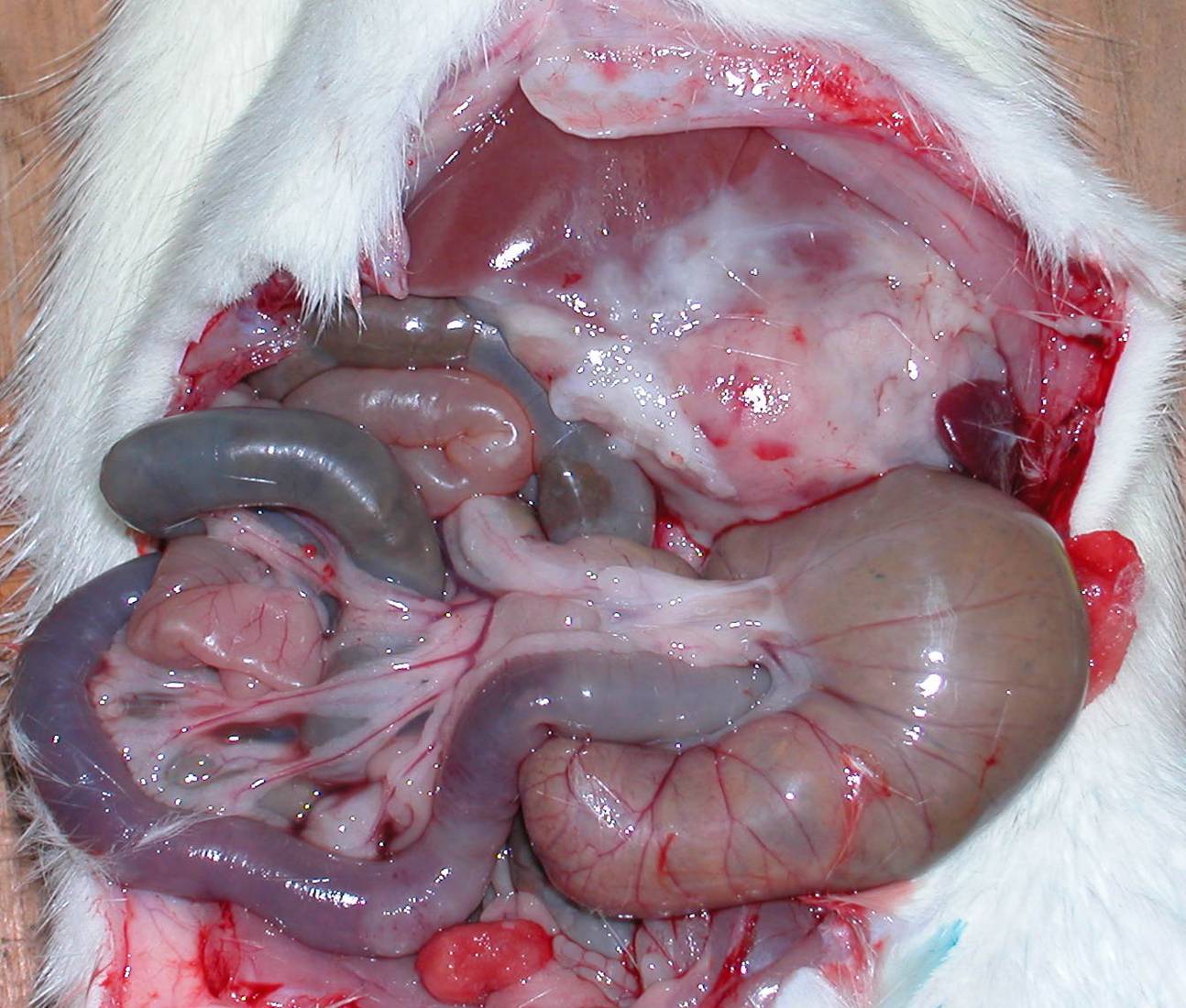

Supplement: Figure S1 — Gross morphology of the peritoneal cavity in a rat model of encapsulating peritoneal sclerosis. Rats were sacrificed 8 days after injection of S. aureus and fibrinogen. A representative rat showing adhesions among the liver, omentum, and intestine. The liver surface was uneven with fusion between lobes. (DOC) [file pone.0044765.s001.doc]
