# Supplementary material for: Fibrin-Induced Epithelial-to-Mesenchymal Transition of Peritoneal Mesothelial Cells as a Mechanism of Peritoneal Fibrosis: Effects of Pentoxifylline
Source: PLoS One. 2012 Sep 13;7(9):e44765. doi: 10.1371/journal.pone.0044765 (PMC3441450; doi:10.1371/journal.pone.0044765)
Supplement: Figure S2 — The Agents were excluded to have cytotoxic effect on PMCs. Bovine serum albumin (BSA, 10 mg/ml), fibrinogen (10 mg/ml), thrombin (0.2 U/ml) and fibrin (by mixing fibrinogen with thrombin at the same concentration) were added into PMC culture for 48 hr. A 3-[4,5-dimethylthiazol-2-yl]-2,5-diphenyltetrazolium bromide (MTT) assay was performed as described (reference 20 in the article). All data are expressed as the means with SEM of 3 experiments conducted in pentaplicate. All agents did not show statistical difference vs. control. (DOC) [file pone.0044765.s002.doc]

**Supporting figure S2. The Agents were excluded to have cytotoxic effect on PMCs.** Bovine serum albumin (BSA, 10 mg/ml), fibrinogen (10 mg/ml), thrombin (0.2U/ml) and fibrin (by mixing fibrinogen with thrombin at the same concentration) were added into PMC culture for 48 hr. A 3-[4,5-dimethylthiazol-2-yl]-2,5-diphenyltetrazolium bromide (MTT) assay was performed as described (reference 20 in the article). All data are expressed as the means with SEM of 3 experiments conducted in pentaplicate. All agents did not show statistical difference vs. control.
