# Supplementary material for: Fibrin-Induced Epithelial-to-Mesenchymal Transition of Peritoneal Mesothelial Cells as a Mechanism of Peritoneal Fibrosis: Effects of Pentoxifylline
Source: PLoS One. 2012 Sep 13;7(9):e44765. doi: 10.1371/journal.pone.0044765 (PMC3441450; doi:10.1371/journal.pone.0044765)
Supplement: Figure S3 — Western blots of cell markers in peritoneal mesothelial cells (PMCs) with various agents. Equal amounts of protein (20 µg per lane) from untreated, bovine serum albumin, thrombin, fibrinogen, or fibrin treated PMCs for 4 hr were resolved, transferred, and blotted for α-SMA, fibronectin, β3 integrin, and GAPDH (A). The relative levels of α-SMA/GAPDH (B), β3 integrin/GAPDH (C), fibronectin/GAPDH (D) were measured by densitometry. C, PMCs without agent; BSA, bovine serum albumin 10 mg/ml; T, thrombin 0.2 U/ml; FG, fibrinogen 10 mg/ml; F, fibrinogen 10 mg/ml mixed with thrombin 0.2 U/ml. *P<0.05 vs. C, # P<0.05 between fibrinogen and fibrin, n = 3. (DOC) [file pone.0044765.s003.doc]

**Supporting figure S3. Western blots of cell markers in peritoneal mesothelial cells (PMCs) with various agents.**

Equal amounts of protein (20 μg per lane) from untreated, bovine serum albumin, thrombin, fibrinogen, or fibrin treated PMCs for 4 hr were resolved, transferred, and blotted for α-SMA, fibronectin, β_3_ integrin, and GAPDH (A). The relative levels of α-SMA/GAPDH (B), β_3_ integrin/GAPDH (C), fibronectin/GAPDH (D) were measured by densitometry. C, PMCs without agent; BSA, bovine serum albumin 10 mg/ml; T, thrombin 0.2U/ml; FG, fibrinogen 10 mg/ml; F, fibrinogen 10 mg/ml mixed with thrombin 0.2U/ml. *P < 0.05 vs. C, # P < 0.05 between fibrinogen and fibrin, n = 3.

A


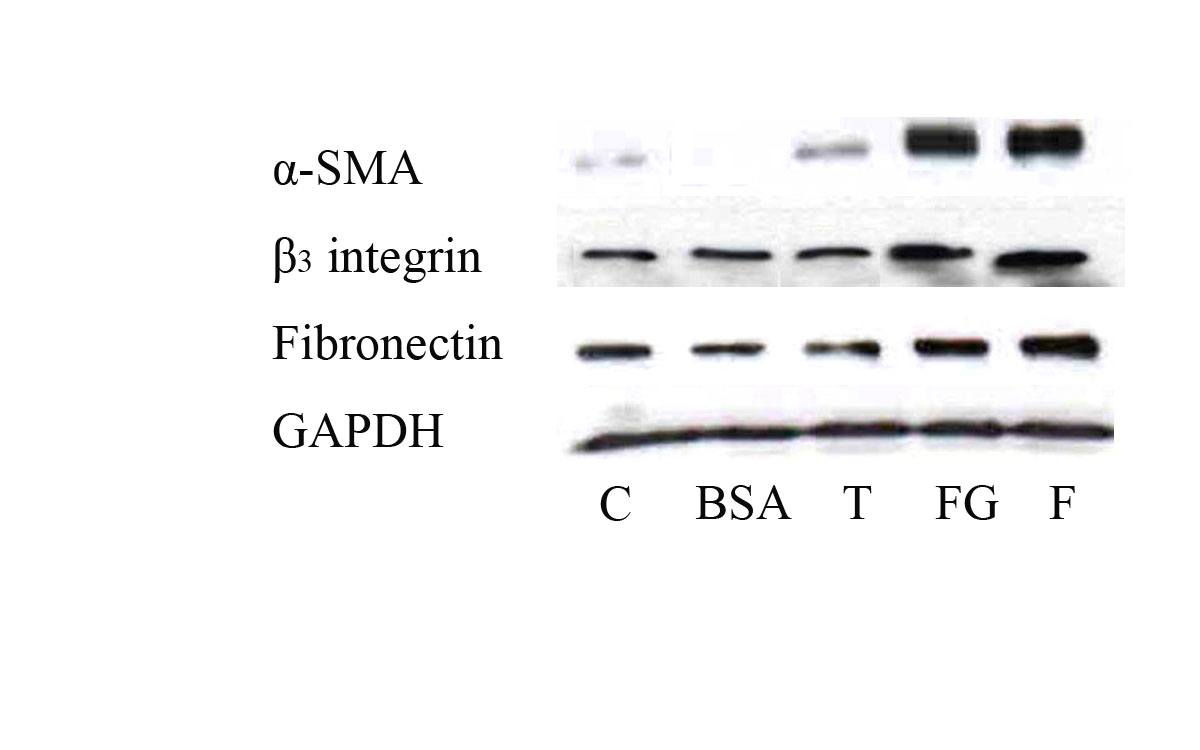


B

C

D
