# Supplementary material for: Fibrin-Induced Epithelial-to-Mesenchymal Transition of Peritoneal Mesothelial Cells as a Mechanism of Peritoneal Fibrosis: Effects of Pentoxifylline
Source: PLoS One. 2012 Sep 13;7(9):e44765. doi: 10.1371/journal.pone.0044765 (PMC3441450; doi:10.1371/journal.pone.0044765)

**Supporting figure S4. Micrographs of tissues from rats injected intraperitoneally with *S. aureus* and fibrinogen.**

The submesothelial compact zones of the parietal peritoneum (A) and liver (B) were stained blue with Masson’s Trichrome stain. Each tissue section was measured at 5 points per microscopic field and 5 fields were recorded. The average thickness of the submesothelial compact zone was determined.

A


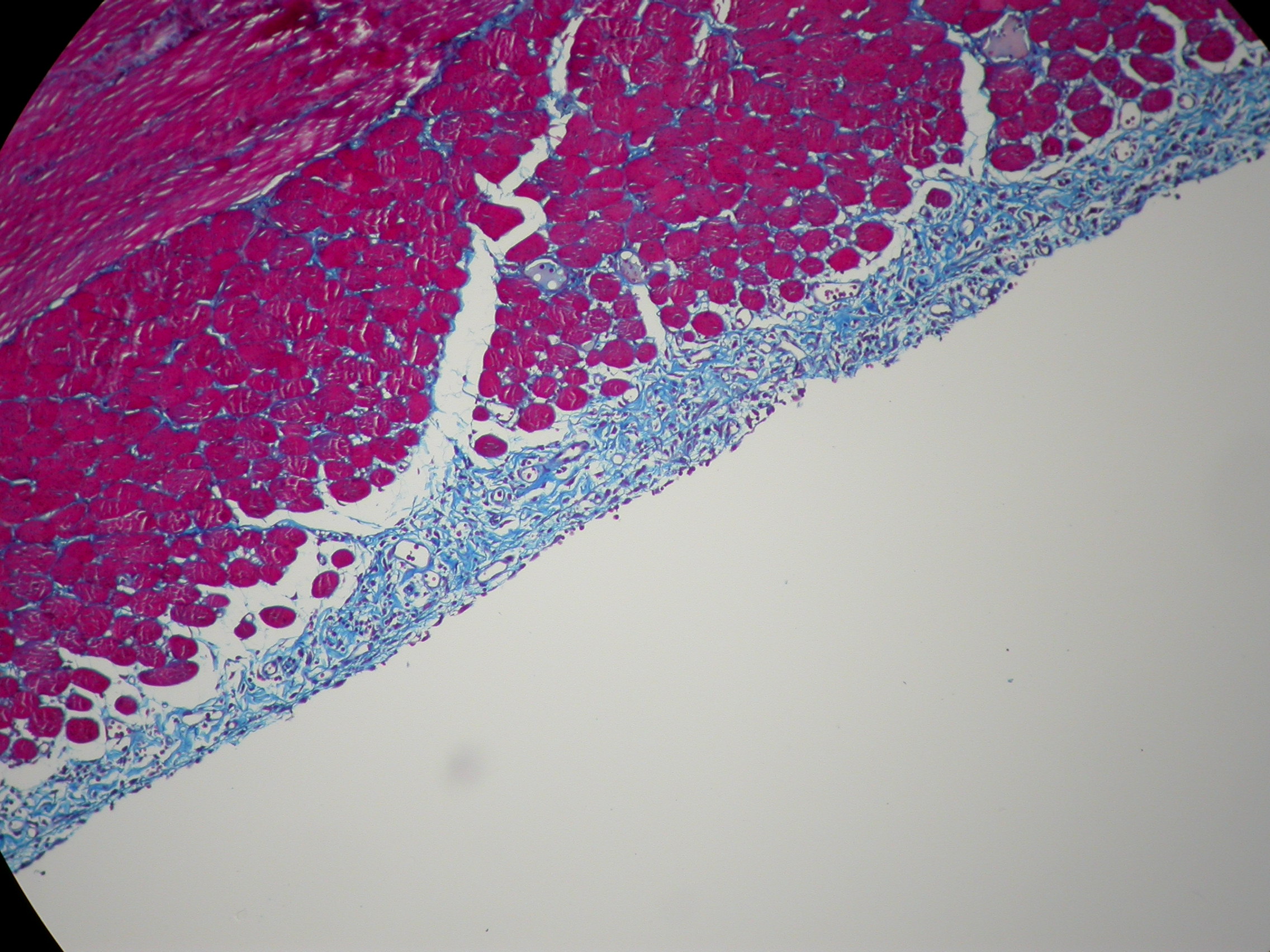


B


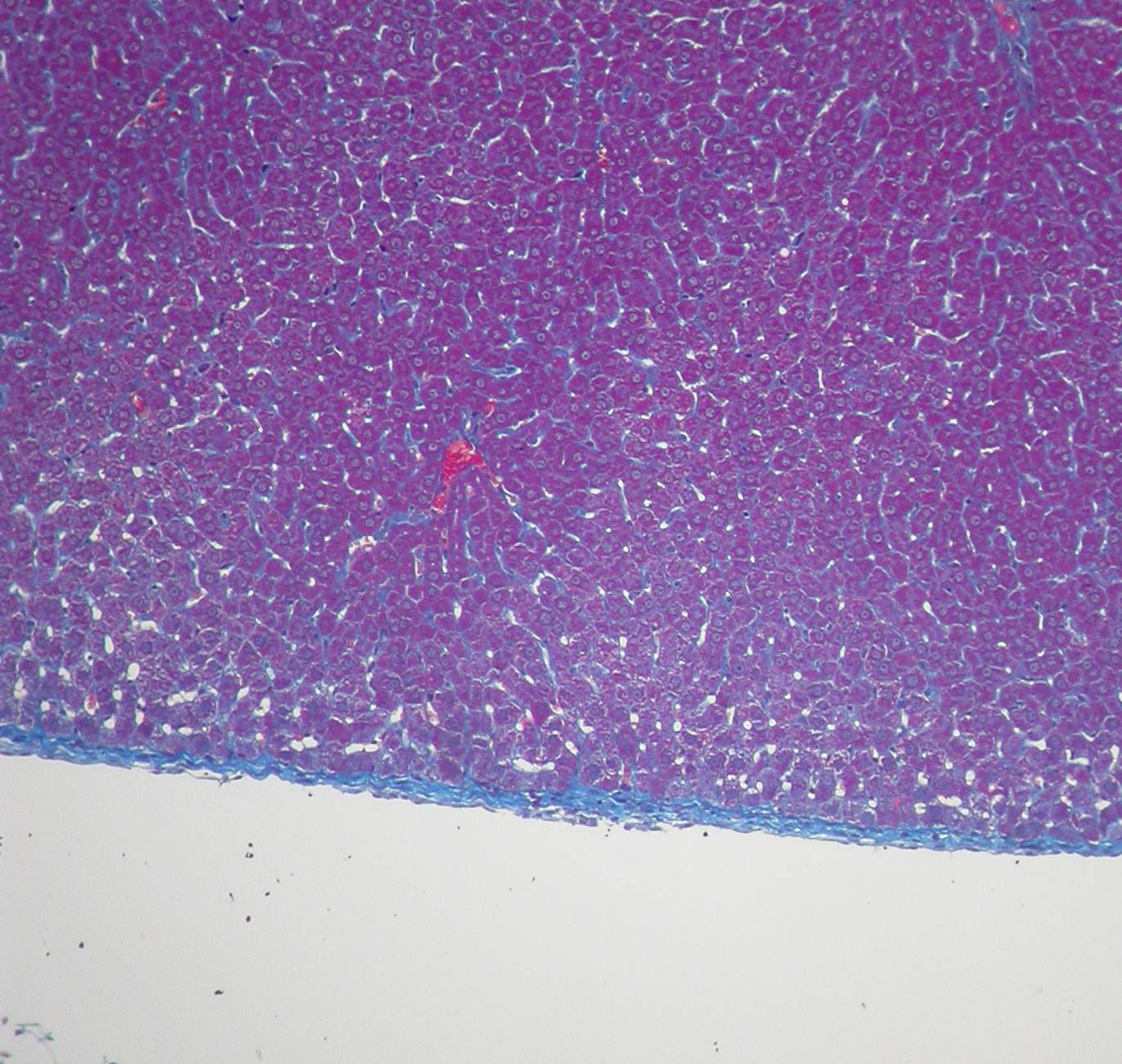

Supplement: Figure S4 — Micrographs of tissues from rats injected intraperitoneally with S. aureus and fibrinogen. The submesothelial compact zones of the parietal peritoneum (A) and liver (B) were stained blue with Masson's Trichrome stain. Each tissue section was measured at 5 points per microscopic field and 5 fields were recorded. The average thickness of the submesothelial compact zone was determined. (DOC) [file pone.0044765.s004.doc]
